# How Landscape Heterogeneity Frames Optimal Diffusivity in Searching Processes

**DOI:** 10.1371/journal.pcbi.1002233

**Published:** 2011-11-03

**Authors:** E. P. Raposo, F. Bartumeus, M. G. E. da Luz, P. J. Ribeiro-Neto, T. A. Souza, G. M. Viswanathan

**Affiliations:** 1 Laboratório de Física Teórica e Computacional, Departamento de Física, Universidade Federal de Pernambuco, Recife, Brazil; 2 Centre d'Estudis Avançats de Blanes, CEAB-CSIC, Blanes, Spain; 3 Departamento de Física, Universidade Federal do Paraná, Curitiba, Brazil; 4 Center for Polymer Studies and Department of Physics, Boston University, Boston, Massachusetts, United States of America; 5 Departamento de Física Teórica e Experimental, Universidade Federal do Rio Grande do Norte, Natal, Brazil; 6 Instituto de Física, Universidade Federal de Alagoas, Maceió, Brazil; University of Michigan and Howard Hughes Med. Inst., United States of America

## Abstract

Theoretical and empirical investigations of search strategies typically have failed to distinguish the distinct roles played by density versus patchiness of resources. It is well known that motility and diffusivity of organisms often increase in environments with low density of resources, but thus far there has been little progress in understanding the specific role of landscape heterogeneity and disorder on random, non-oriented motility. Here we address the general question of how the landscape heterogeneity affects the efficiency of encounter interactions under global constant density of scarce resources. We unveil the key mechanism coupling the landscape structure with optimal search diffusivity. In particular, our main result leads to an empirically testable prediction: enhanced diffusivity (including superdiffusive searches), with shift in the diffusion exponent, favors the success of target encounters in heterogeneous landscapes.

## Author Summary

Understanding how animals search for food is crucial for animal ecology. Although much has been learned about the main aspects of the so-called foraging problem, some important questions still remain unanswered. In this work we address the issue of the relevance of heterogeneity in the resources distribution to efficient animal foraging behavior. Our results unveil the key mechanism coupling landscape heterogeneity dynamics with optimal search diffusivity. Indeed, although the effect of (global) resource density on animal foraging behavior is well documented, much less has been known about how spatiotemporal landscape heterogeneity affects the efficiency of encounter interactions by foraging organisms. In this sense, we propose a new empirically testable theoretical prediction on the dynamics (e.g. diffusion exponent) of foraging organisms in heterogeneous environments. We also show that the conditions in which Lévy strategies are optimal are much broader than previously considered.

## Introduction

The random search problem has lately received a great deal of attention [1], [2]. This is partly due to its broad interdisciplinary range of applications, which include, e.g., enhanced diffusion of regulatory proteins while “searching” for specific DNA spots [3], [4] and the finding of binding sites on transmembrane proteins by neurotransmitters in the brain [5]. Recently, this problem has also found interesting connections with human mobility and related topics [6]–[9].

A classical context in which the random search problem has been applied in the last four decades is animal foraging [1], [2], [10]–[27], with the searcher (i.e. forager) typically represented by an animal species in quest of target sites (prey, food, other individuals, shelter, etc.) in a search landscape.

Among the most studied random walk models proposed as plausible search strategies, we cite correlated random walks [12], [28], [29], Lévy flights and walks [13]–[17], [19], [20], [24], [25], [27], [30]–[39], intermittent walks [40]–[46], and composite Brownian walks [47], [48]. In particular, Lévy random searchers, with probability distribution of step lengths 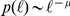, for 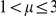, have successfully explained [34] the emergence of optimal searches in landscapes with randomly and scarcely distributed target sites. On the other hand, when resources are plentiful Lévy strategies are unnecessary [34], and efficient Brownian optimal searches may arise with, e.g., a Poisson-like exponential distribution 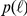 [24], [25]. Lévy flights and walks have been also shown to be relevant in several other contexts [1], such as in proteins searching for specific DNA sites [49], in which the optimal Lévy mechanism emerges directly from the underlying physics of the problem (polymer scaling theory in three dimensions).

In the regime of low density of resources of the random search problem, two limiting situations have been extensively considered [34]: (i) non-destructive searches, in which the searcher always departs from a position at the vicinity of the last target found with unrestricted revisits; and (ii) destructive searches, in which, once found, the target becomes inaccessible to future visits, so that the starting point of the searcher is, on average, faraway from all targets. In the former case the maximum efficiency is achieved [34] for 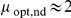 (a “compromise” superdiffusive solution), whereas in the latter 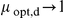 (ballistic motion). It is important to observe, nevertheless, that by varying the searcher's starting point [44], [48] or the degree of target revisitability or temporal regeneration [50], [51], intermediate values of the optimal Lévy exponent arise, 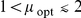.

It is also interesting to comment on the effect of an energy cost function on the efficiency of search strategies. Indeed, as reported in [50], [51], the range of 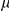-values associated with search paths in which the net energy gain (the balance between the energy income due to the finding of targets and the energy cost of the search process itself) remains always positive is actually limited. In such a case, low values of 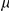 giving rise to very large search jumps might not be acceptable, since they imply a high energy cost, with intermediate values of 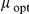 emerging as the best strategy. In addition, we also refer to the study reported in [52] in which exact results for the first passage time and leapover statistics of Lévy flights are presented. In this case, the targets might not be always detected, being thus overshoot by jumps whose length distribution displays infinite variance.

Despite the intense progress in the fields of random searches and animal foraging, a number of relevant issues still remain open. A particularly important one is to understand the coupling mechanism between landscape spatiotemporal dynamics and efficient search motility, when resources are scarce and environmental information is limited. In this sense, the pervasiveness of different animal search strategies is expected to strongly depend on a few but essential features of actual landscapes. For instance, targets distributions in realistic search processes usually present heterogeneous properties through time and space, such as diverse degrees of temporal regeneration and spatial aggregation [26], [53], [54]. Although the effect of (global) resource density on animal foraging behavior is well documented [25], [26], [37], [42], [55], much less is known about how spatiotemporal landscape heterogeneity dynamics affects the target revisitability and/or searcher-to-targets distances, both known to be key properties to optimize perception-limited searches [44], [48], [50], [51]. Thus, a mechanistic understanding of how and which landscape features are related to search efficiency should be a relevant step towards a comprehensive view of animal foraging behavior.

Here we address the question of how the landscape heterogeneity influences the encounter success and search efficiency under conditions of constant (global) density of scarce resources. We develop a random search model in which diverse degrees of inhomogeneities are considered by introducing fluctuations in the starting distances to target sites. We thus ask what happens to the optimal search strategy in an heterogeneous landscape, as the searcher's initial distances to the targets fluctuate along the search. We answer to this query qualitatively for the general case and quantitatively for Lévy random searches in particular, in the constant density regime of scarce resources. In patchy or aggregated landscapes, we find that enhanced diffusivity (including superdiffusive strategies) favors the encounter of targets and the success of foraging. Eventually, for strong enough fluctuations in the starting distances to nearby targets a crossover to ballistic strategies might emerge.

These predictions are empirically testable through feasible experiments which investigate the dynamics (e.g. diffusion exponent) of foraging organisms in specially designed low-density environments of controlled heterogeneity.

## Materials and Methods

### Distributions of starting positions: General considerations

We consider a random search model in which diverse degrees of landscape heterogeneity are taken into account by introducing fluctuations in the starting distances to target sites in a one-dimensional (1D) search space, with absorbing boundaries separated by the distance 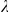. Every time an encounter occurs the search resets and restarts over again. Thus, the overall search trajectory can be viewed as the concatenated sum of partial paths between consecutive encounters. The targets' positions are fixed – targets are in fact the boundaries of the system. Fluctuations in the starting distances to the targets are introduced by sampling the searcher's departing position after each encounter from a probability density function (pdf) 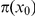 of initial positions 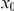. Importantly, 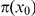 also implies a distribution of starting (a)symmetry conditions regarding the relative distances between the searcher and the boundary targets.

This approach allows the typification of landscapes that, on average, depress or boost the presence of nearby targets in the search process. Diverse degrees of landscape heterogeneity can thus be achieved through suitable choices of 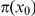.

For example, a pdf providing a distribution of nearly symmetric conditions can be assigned to a landscape with a high degree of homogeneity in the spatial arrangement of targets. In this sense, the mentioned destructive search represents the fully symmetric limiting situation, with the searcher's starting location always equidistant from all boundary targets. On the other hand, a distribution 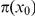 which generates a set of asymmetric conditions is related to a patchy or aggregated landscape. Indeed, in a patchy landscape it is likely that a search process starts with an asymmetric situation in which the distances to the nearest and farthest targets are very dissimilar. Analogously, the non-destructive search corresponds to the highest asymmetric case, in which at every starting search the distance to the closest (farthest) target is minimum (maximum). Finally, a pdf 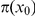 giving rise to an heterogeneous set of initial conditions (combining symmetric and asymmetric situations) can be associated with heterogeneous landscapes of structure in between the homogeneous and patchy cases.

More specifically, the limiting case corresponding to the mentioned destructive search can be described by the pdf with fully symmetric initial condition,
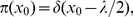
(1)where 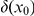 denotes Dirac 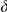-function. This means that every destructive search starts exactly at half distance from the boundary targets. In this context, it is possible to introduce fluctuations in 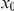 by considering, e.g., a Poisson-like pdf [56] exponentially decaying with the distance to the point at the center of the search space, 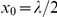:
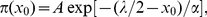
(2)where 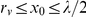, with 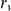 the “radius of vision” of the searcher (see below), 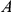 the normalization constant, and 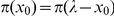 due to the symmetry of the search space.

On the other hand, the highest asymmetric non-destructive limiting case is represented by
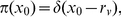
(3)so that every search starts from the point of minimum distance in which the nearest target is undetectable, 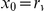. Similarly, fluctuations in 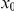 regarding this case can be introduced by considering a Poisson-like pdf decreasing with respect to the point 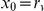:
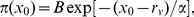
(4)where 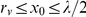, 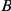 is a normalization constant, and 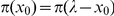. In Eqs. (2) and (4), the parameter 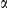 controls the range and magnitude of the fluctuations. Actually, the smaller the value of 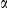, the less disperse are the fluctuations around 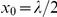 and 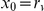 in Eqs. (2) and (4), respectively.

### Random search model in 1D

When looking for boundary target sites in a 1D interval, the searcher's step lengths 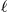 are taken from a *general* pdf 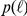. At each step the probabilities to move to the right or to the left are equal. We define the “radius of vision” 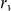 as the distance below which a target becomes detectable by the searcher. Thus, if the targets are located at the boundary positions 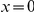 and 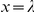, the search keeps on as long as the walker's position lies in the range 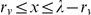. Here we are interested in searches in environments scarce in targets, i.e. for 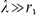. In this case, leaving the present position to look randomly for targets should occur much more frequently than simply detecting a site in the close vicinity, a regime favored when targets are plentiful.

Suppose initially that, as a target is found, the search always restarts from the same position 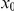 in the interval 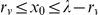. As discussed, the highest asymmetric (non-destructive) and fully symmetric (destructive) cases correspond respectively to setting 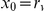 (or 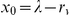, due to symmetry) and 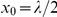. After the encounter of a statistically large number of targets, the efficiency of the search, 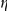, is evaluated [34] as the ratio of the number of sites found to the total distance traversed by the searcher. Since this distance is equal to the product of the number of encounters and the average distance traveled between consecutive findings, 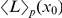, then 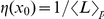.

Consider now that, instead of always departing from the same location after an encounter, the searcher can restart from any initial position 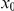 in the range 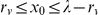, chosen from a pdf 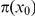. The fluctuating values of 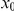 imply a distribution of 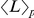 values. Since searches starting at 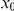 are statistically indistinguishable from searches starting at 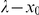 (in both cases the closest and farthest targets are at distances 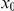 and 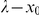 from the starting location), the symmetry of the search space regarding the position 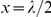 implies 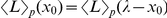. The average efficiency thus becomes
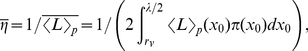
(5)where 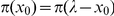 due to the above mentioned symmetry.

To study the effect of fluctuations in the starting distances of a searcher, we note that the exact average distance 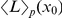 in Eq. (5) can be formally expressed [57], [58] as
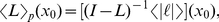
(6)where the integral operator 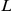 acts as follows:
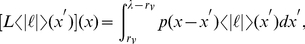
(7)and 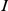 and 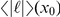 are, respectively, the unity operator and the average length of a single step starting at 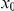. Specifically, we can write for a general pdf 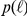


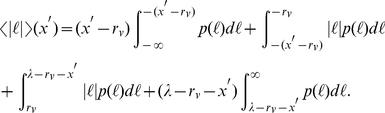
(8)The second and third integrals above represent steps to the left and to the right which are not truncated by the encounter of a target site at the boundaries; the first and last ones concern steps truncated by the detection of the targets at 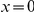 and 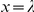, respectively (what actually happens at 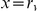 and 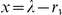, due to the searcher's “radius of vision”).

Despite the formal aspect of Eq. (6), the numerical calculation of 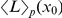 with a given 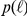 can be performed by discretizing [57], [58] the search interval 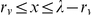, i.e. 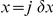, with 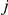 integer and 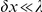. In this procedure, integrals are approximated by summations, and so on.

In the next section, we use this model to study the role of landscape heterogeneity on the search efficiency and diffusivity. The presented analysis is qualitative for the general case and quantitative for Lévy random searches.

## Results

### Efficient search strategies with a general pdf of step lengths

Consider, first, the limiting case with no fluctuation in the starting distances. The underlying mechanisms of efficient searches with asymmetric and symmetric initial conditions are fundamentally distinct. In the fully symmetric (destructive) case 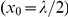 the closest sites are located at equal initial distances 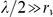 from the searcher in the low-density regime. Thus, for a general distribution of step lengths 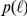 characterized by a set of parameters 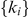, the one 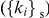 that leads to the largest efficiency 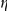 must present the fastest possible diffusivity in order to reach these faraway targets. For example, in the case of the single-parameter power-law pdf 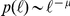, 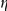 is maximized with ballistic strategy [34]: 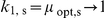.

**Figure 1:**
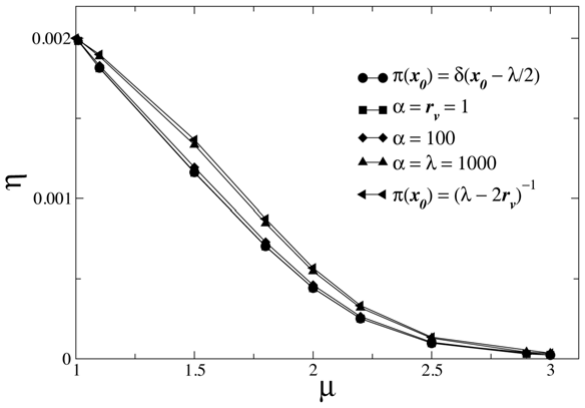
Robustness of the ballistic optimal search strategy with respect to fluctuations in the distances to faraway target sites. In the case of Lévy random searchers, for 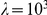 and 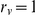, the average search efficiency 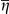, Eq. (5), is always highest for 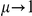 (ballistic dynamics), for any value of the parameter 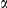 of the Poissonian fluctuations around the maximum allowed distance, 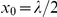, Eq. (2). Cases with uniform and without any (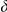-function) fluctuation are also shown. Solid lines are a visual guide.

**Figure 2:**
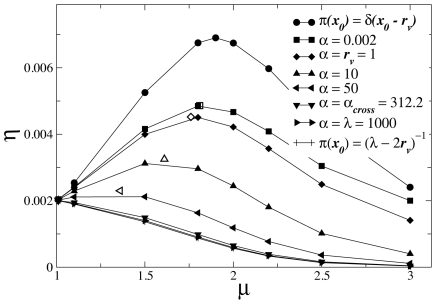
Shift in the optimal search strategy towards an enhanced superdiffusive dynamical regime, as landscapes with distinct degrees of heterogeneity are considered. For Lévy random searchers, using 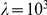 and 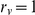 (solid symbols), the average search efficiency 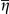, Eq. (5), is maximized for smaller 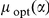 (faster diffusivity) in the case of wider (larger-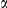) Poissonian fluctuations in the distances to nearby target sites, Eq. (4). Cases with uniform and without any (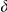-function) fluctuation are also shown (solid lines are a visual guide). Empty symbols locate the maximum 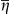 obtained from the condition 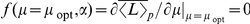. For strong enough fluctuations, with 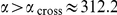, a crossover to ballistic dynamics (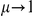) emerges.

In contrast, in the highest asymmetric (non-destructive) situation 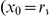 or 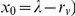 the most efficient search must compromise between performing large steps to access the farthest site and sweeping in detail at the vicinity of the closest site. In the parameter space, this solution, related to a set 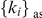, displays intermediate diffusivity between normal (Brownian) and the fastest possible one, assigned to the set 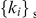. In the same example, this implies [34] 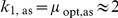, in contrast with Brownian diffusion resulting from 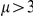 (see Figs. 1 and 2).

When the starting positions are not fixed, heterogeneous landscapes with stronger fluctuations in the distances to nearby targets lead to optimal search strategies with faster dynamics (enhanced diffusivity). The arguments giving rise to this general conclusion are as follows.

**Figure 3:**
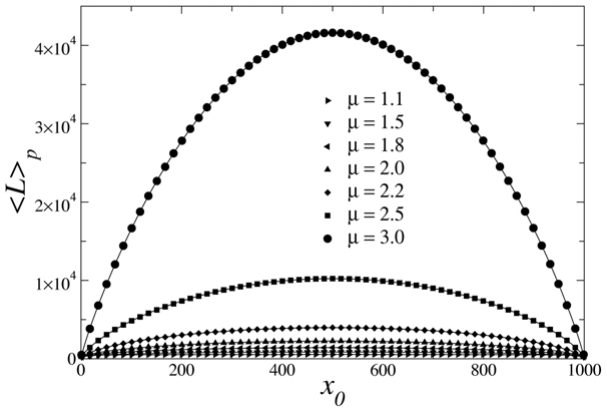
Nice adjustment of the average distance 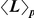
 **traversed between consecutive findings by a Lévy random searcher starting at position** 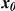 **.** Results obtained by numerical discretization of Eq. (6) (solid lines) and multiple regression (symbols), for 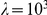 and 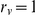.

On one hand, sampling starting positions around 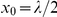 corresponds to introduce fluctuations in the initial distances to the faraway boundary targets in the low-density regime, as discussed. In this case, we expect that starting positions far away from 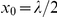 are chosen with smaller probabilities. This implies a decreasing pdf 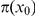 from 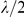 to 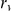, such as found in Eq. (2). Consequently, both 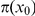 and 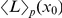 increase monotonically from 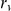 to 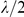 (Fig. 3). The most relevant contribution to the product 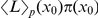 in Eq. (5) thus comes from positions near 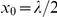. No qualitative difference is expected to occur between 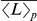 and 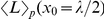, indicating that searches with fully symmetric (fixed) initial condition and those comprising fluctuations in the faraway targets present similar optimal dynamics, related to the set 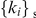, namely ballistic, if supported by 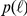.

On the other hand, in the asymmetric case fluctuations in the starting distances to the nearby boundary target can be introduced by a decreasing pdf 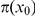 from 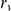 to 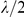, such as in Eq. (4). Therefore, as 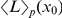 increases and 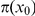 diminishes, the initial position associated with the most relevant contribution to 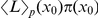 in Eq. (5) crosses over to somewhere in between 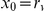 and 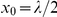. Indeed, the slower 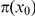 decays, the larger such position becomes. As a consequence, the asymmetric optimal set 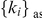 in the absence of fluctuations might give away the role of the most efficient search strategy to some other intermediate compromising solution 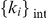, which is closer to the symmetric set 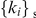 in the parameter space and, therefore, presents enhanced dynamics (e.g., a larger diffusion exponent). Eventually, for some proper choice of 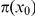 encompassing strong fluctuations with large weight near 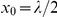, the justification for such compromising solution might even fade away, so that 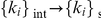, with strategies of fastest possible diffusivity becoming optimal. In this uttermost case fluctuations lose their local character, and a crossover from superdiffusive to ballistic search behavior may take place.

We observe that the above rationale should also apply, at least qualitatively, to searches in higher-dimensional spaces. In this situation, as the search path can be approximated by a sequence of nearly rectilinear moves, the general qualitative features of 1D random searches usually hold true in higher dimensions [34], [39]. Nevertheless, the finding of targets in 2D and 3D occurs with considerably lower probability, since the extra spatial directions yield a larger exploration space, resulting in lower encounter rates and search efficiencies. The impact of target spatial fluctuations on high-dimensional search strategies should also reduce [39]. We can thus conclude that, beyond representing the realistic exploration space of some animal species [27], the 1D analysis presented here is also useful in establishing upper limits for the influence of landscape heterogeneities in random searches. Therefore, the understanding of animal foraging behavior in 2D and 3D, as well as other practical realizations of the random search problem, might also benefit from the present results.

We next apply the above arguments, valid for a general pdf 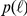, to the particular case of Lévy random searchers.

### Lévy searches in heterogeneous landscapes

We now specifically consider a random searcher with step lengths chosen from the pdf
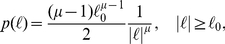
(9)and 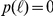 otherwise, with 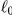 representing a lower cutoff length. We assign a “negative step length” 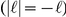 if the searcher moves to the left and take 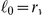 for simplicity. Equation (9) for 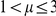 corresponds to the long-range asymptotical limit of Lévy 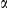-stable distributions with index 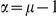, characterized by the existence of rare, large steps alternating between sequences of many short-length jumps [13], [14], [16]. As its second moment diverges the central limit theorem does not hold, and anomalous (superdiffusive) dynamics governed by the generalized central limit theorem takes place. Indeed, Lévy random walks and flights are related to a Hurst exponent [13], [14] 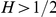, with ballistic dynamics in the case 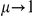, whereas diffusive behavior (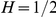) emerges for 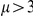. For 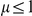 pdf (9) is not normalizable and 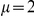 corresponds to the Cauchy distribution.

The search path eventually comprises truncated steps due to the encounter of targets, so that the power-law decay of Eq. (9) cannot extend all the way to infinity, thus implying an effective truncated Lévy distribution [59]. In spite of this, in the regime 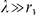 the search should retain the most relevant properties of a non-truncated Lévy walk to a considerable extent. Indeed, the ratio 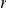 of the number of truncated steps to the non-truncated ones, essentially equal to the inverse of the average number of steps performed between consecutive targets, is given by 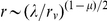 and 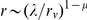, for 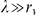, in the highest asymmetric (non-destructive) and fully symmetric (destructive) cases, respectively [34], [57], [58]. Thus, except for 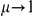 ballistic walks, one has that 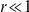 if 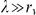. Further, the justification for truncated distributions also arises naturally in the context of animal foraging since directional persistence due to scanning is likely to be broken at the finding of targets [19]. Indeed, infinitely long rectilinear paths are not allowed for searching organisms.

By inserting Eq. (9) into Eqs. (6) and (7), we numerically calculate 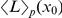 through the discretization of the search space (see previous section). Results are displayed in solid lines in Fig. 3. Notice first the presence of the symmetry 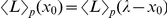 discussed above. In the absence of fluctuations in the initial distances, the existence of a maximum efficiency with an intermediate exponent 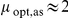 (see Fig. 2) for searches starting at fixed 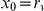 (highest asymmetric condition) can be understood as follows: strategies with 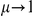 might access the farthest target at 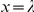 in a ballistic way after a small number of very large steps, implying a large 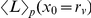 and low efficiency; in contrast, searches with 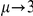 tend on average to find the closest site at 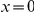 after a great number of small steps, also giving rise to a large 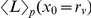; the efficient compromise between these two trends, leading to the lowest 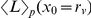 and maximum 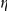, is therefore represented by a strategy with an intermediate value, 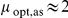.

In the presence of fluctuations in the starting distances, the integral (5) must be evaluated. Although the explicit expression for 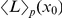, Eq. (6), is not known up to the present, a multiple regression can be successfully performed,
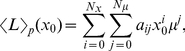
(10)as indicated by the nice adjustment shown in Fig. 3, obtained with 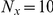 and 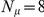. Thus, the integral (5) can be done using Eqs. (2), (4) and (10), with results displayed in Figs. 1 and 2 for several values of the parameter 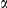.

By considering fluctuations in the starting distances to faraway targets through Eq. (2), we notice in Fig. 1 that the efficiency is qualitatively similar to that of the fully symmetric condition, Eq. (1), in agreement with the general arguments of the previous section. Indeed, in both cases the maximum efficiency is achieved as 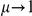. For 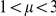 the presence of fluctuations only slightly improve the efficiency. These results indicate that ballistic strategies remain robust to fluctuations in the distribution of faraway targets.

On the other hand, fluctuations in the starting distances to nearby targets, Eq. (4), are shown in Fig. 2 to decrease considerably the search efficiency, in comparison to the highest asymmetric case, Eq. (3). In this regime, since stronger fluctuations increase the weight of starting positions far from the target at 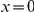, the compromising optimal Lévy strategy displays enhanced superdiffusion, observed in the location of the maximum efficiency in Fig. 2, which shifts from 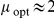, for the delta pdf and Eq. (4) with small 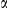, towards 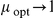, for larger 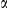 (slower decaying 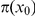). Indeed, both the pdf of Eq. (4) with a vanishing 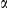 and Eq. (3) are very acute at 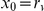. It is also worth noticing that a lower 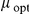 is related to a larger Hurst exponent [1], [13], [14], and therefore to a larger diffusion exponent, as argued in the previous section.

As even larger values of 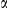 are considered, fluctuations in the starting distances to the nearby target become non-local, and Eq. (4) approaches the 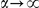 limiting case of the uniform distribution, 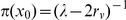 (see Fig. 2). In this situation, search paths departing from distinct 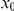 are equally weighted in Eq. (5), so that the dominant contribution to the integral (and to the average efficiency 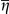 as well) comes from search walks starting at positions near 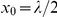. Since for these walks the most efficient strategy is ballistic, a crossover from superdiffusive to ballistic optimal searches emerges, induced by such strong fluctuations. Consequently, the efficiency curves for very large 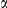 (Fig. 2) are remarkably similar to that of the fully symmetric case (Fig. 1).

We can quantify this crossover shift in 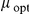 by defining a function 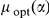 that identifies the location in the 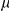-axis of the maximum in the efficiency 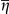, for each curve in Fig. 2 with fixed 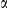. As discussed, eventually a compromising solution with 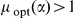 cannot be achieved, and an efficiency function 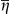 monotonically decreasing with increasing 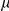 arises for 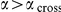. In this sense, the value 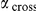 for which such crossover occurs marks the onset of a regime dominated by ballistic optimal search strategies.

**Figure 4:**
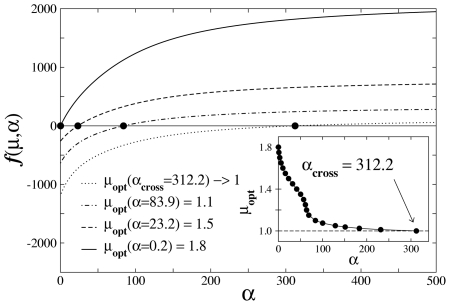
Determination of the optimal search strategy of Lévy random searchers with Poissonian fluctuations in the distances to nearby targets, **Eq. (4)**. The condition 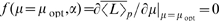, for 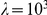 and 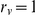, provides the optimal Lévy exponent, 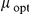, associated with the strategy of maximum average efficiency. Inset: since strategies with 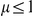 are not allowed (non-normalizable pdf of step lengths), the highest efficiency is always obtained for 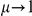 as fluctuations with 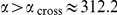 are considered, marking the onset of a regime dominated by ballistic optimal search dynamics.

The value of 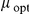 for each 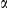 can be determined from the condition 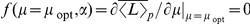, so that, by considering Eqs. (4), (5) and (10),
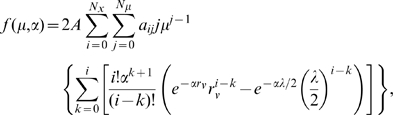
(11)with 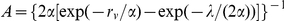. Solutions are displayed in Fig. 4 and also in Fig. 2 as empty symbols, locating the maximum of each efficiency curve. In addition, the crossover value can be determined through 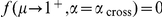. In the case of pdf (4), we obtain (Fig. 4) 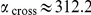 for 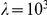 and 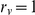 (regime 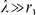).

We also note that the scale-dependent interplay between the target density and the range of fluctuations implies a value of 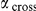 which is a function of 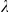. For instance, a larger 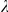 (i.e., a lower target density) leads to a larger 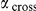 and a broader regime in which superdiffusive Lévy searchers are optimal. Nevertheless, the above qualitative picture should still hold as long as low target densities are considered.

Moreover, since ballistic strategies lose efficiency in higher dimensional spaces [44], it might be possible that in 2D and 3D the crossover to ballistic dynamics becomes considerably limited. In spite of this, enhanced superdiffusive searches, with 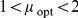, should still conceivably emerge due to fluctuations in higher-dimensional heterogeneous landscapes.

From these results we conclude that, in the presence of Poissonian-distributed fluctuating starting distances with 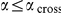, Lévy search strategies with faster (enhanced) superdiffusive properties, i.e. 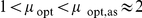, represent optimal compromising solutions. In this sense, as local fluctuations in nearby targets give rise to landscape heterogeneity, Lévy searches with enhanced superdiffusive dynamics actually maximize the search efficiency in aggregate and patchy environments. On the other hand, for strong enough fluctuations with 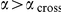, a crossover to the ballistic strategy emerges in order to access efficiently the faraway region where targets are distributed. These findings are in full agreement with the general considerations discussed in the previous section.

At last, to further test the robustness of these results we have also considered the power-law distribution of starting positions, 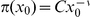, with 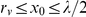, 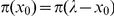, and 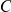 as the normalization constant. Differently from distributions (2) and (4), the long tail in this pdf confers self-affine scale-invariant properties over a long spatial range in the low-density regime, 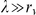. The evidence of scale-free distributions of targets has been reported in the context of animal foraging, e.g. in [24]. In the present analysis we have essentially verified all the general features previously discussed. In particular, all strategies with 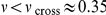 are ballistic, with compromising superdiffusive solutions arising for 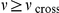.

## Discussion

The effect of limited resources on animal motility is well documented in ecology. Scarcity coming from resource competition is known to induce higher dispersal rates [60], [61] and larger home ranges [62], [63]. Habitat fragmentation also reshapes dispersal kernels, often increasing dispersal distances [64]. In the context of foraging behavior, the role of (global) resource density has been considerably investigated, with strong evidence pointing to shifts from Brownian to superdiffusive search strategies as animals move from high to low productive areas. Examples range from microorganisms [37] to large marine predators [25], [26], [55]. In contrast, much less is known about the influence of heterogeneity in the resource distribution on the foraging success.

Most theoretical efforts relying on core random search theory have by far provided only a limited approach to the issue of optimal searches, since they mostly assume oversimplified landscapes [2], [40]. Nonetheless, a few simulation studies have addressed the effect of environmental heterogeneity, including target motion, on encounter success for different searcher types [19], [24], [39], [65], [66]. These works give support to the hypothesis that search processes are linked to target distributions and dynamics, thus agreeing with our results in that the optimal strategy can actually change, e.g. from superdiffusive to ballistic motion, depending on the landscape heterogeneity. In a more recent example, it was shown [65] that Lévy optimal foragers can be evolutionarily optimal in heterogeneous environments, for suitable details of the simulations and definition of efficiency. Our work advances on this topic by pinpointing a very general mechanism which seems essential to understand previous simulation results [19], [24], [39], [65].

By comprehensively describing the key mechanism coupling landscape dynamics and search diffusivity, we have shown that statistical fluctuations in the set of initial search conditions play a crucial role for determining which strategy is optimal. The presence of such fluctuations sets a clear basis for the non-universality of search patterns, and shows that enhanced diffusivity (including superdiffuse strategies) favors random encounter success in patchy and aggregated landscapes. As a consequence, the foraging conditions in which Lévy strategies appear as optimal are much broader than previously suggested [40], [44]–[46].

In dynamic and complex landscapes with scarcity of resources neither ballistic nor Lévy strategies should be considered as universal (see, e.g., [45], [46]), since realistic fluctuations in the targets distribution may induce switches between these two regimes. This observation has been confirmed by recent empirical results [25], [27], showing that foragers in the wild do not exhibit movement patterns that can be approximated, at all times, by Lévy, ballistic or exponential models. Nevertheless, the relevant finding is that in the low-density regime superdiffusive Lévy strategies remain as the optimal solution in a broad range of heterogeneous landscape conditions, with the optimal exponent 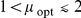 dependent on specific environment properties. Crossovers between superdiffusive and ballistic strategies may also emerge depending on whether strong target spatial fluctuations are local or not, and if they depress or boost the presence of nearby targets. For instance, recent data on a species of jellyfish have reported [27] on Lévy flight foraging strategies with optimal index as low as 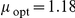. Moreover, studies on marine predators have also found [24] small values as 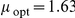. Such rather fast, enhanced superdiffusion (with respect to 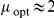) suggests the occurrence of foraging activity in a highly dynamic and heterogeneous landscape, as it is clearly the case for marine prey landscapes [25], [26], [67].

In the present work, the question of how the landscape heterogeneity affects the search efficiency in encounter interactions is addressed under conditions of constant global density of scarce resources. In such conditions we predict that efficient strategies with larger diffusion exponents (including superdiffusive ones) should arise, as heterogeneous environments with wider distributions of starting distances between the foraging organism and the nearby targets are considered. Similarly to what occurs in homogeneous landscapes [42], we do not expect density fluctuations in the scarcity regime to modify optimal Lévy solutions *per se*, but only to the extent that fluctuations in density modify the initial searcher-to-targets distances. In other words, provided that the asymmetry in the searcher-to-targets distances is maintained as density changes, optimal Lévy strategies should result insensitive to target density fluctuations. This means that for a Lévy searcher is less important to have advanced knowledge of the density than of the relative positions of the targets. Clearly, robustness to changes in environmental parameters (i.e. density) should be considered as an advantage in non-informed optimal search solutions [42].

If we acknowledge the presence of selective pressures responsible for the evolution and maintenance of non-oriented motility in organisms [68], our results lead to a neat empirically testable prediction: patchy and heterogeneous landscapes should promote the emergence of enhanced diffusivity and compromising optimal Lévy strategies. Even though the empirical inference of large scale movement patterns from heterogeneity properties of the landscape is a difficult task [26], specifically designed and controlled large scale experiments are feasible in the laboratory [68]–[71] and even in the field [54].

We hope the present study might shed light on unsettled issues related to the efficiency and associated dynamics of organisms performing random searches. Besides the well documented dependence of search efficiency on resource density [25], [26], [34], [37], [55], our results suggest another relevant aspect of non-universal random search behavior: landscape heterogeneity frames optimal diffusivity.
